# Menstrual tampons and vaginal pessaries: regulation of intravaginal medical devices by the US FDA

**DOI:** 10.3389/frph.2023.1224421

**Published:** 2023-09-19

**Authors:** Colin M. Pollard

**Affiliations:** Consultant, Washington, DC, United States

**Keywords:** menstrual tampons, vaginal pessaries, medical device, regulation, food and drug administration, 510(k) premarket notification

## Abstract

Catamenial products like menstrual tampons for managing menses and vaginal pessaries for managing urinary incontinence and uterine prolapse are products that can be inserted and removed from the vagina repeatedly over a woman's lifetime. In the United States (US), these products are considered to be medical devices and are regulated by the Center for Devices & Radiological Health (CDRH) of the Food and Drug Administration (FDA). As such, they are subject to both premarket and postmarket regulatory controls. Both tampons and pessaries have a long history of safe and effective use, and FDA applies a risk-based approach to both premarket entry as well as continued postmarket regulatory controls. Practicing clinicians are often the initial source of ideas for medical device improvements. This article is intended to help such clinicians to understand the regulatory challenges faced by development teams who seek to introduce these kinds of products to the US market. It explains FDA's risk-based classification of medical devices and the 510(k) premarket notification as the primary regulatory mechanism for market entry. It also highlights key FDA guidance documents and encourages early engagement with FDA when appropriate.

Menstrual tampons and vaginal pessaries are medical devices that are inserted into the vagina to achieve their effect. Women have used menstrual tampons for decades to manage menstrual bleeding. Likewise, women have used vaginal pessaries for decades as a nonsurgical option for the management of urinary incontinence and pelvic organ prolapse. These devices can be inserted intravaginally and removed, repeatedly, for years. In general, FDA considers both tampons and pessaries to be safe and effective medical devices. Under a statutory risk-based classification scheme, these products are regulated by the Center for Devices and Radiological Health (CDRH), part of the Food and Drug Administration (FDA), to ensure their continued safety and effectiveness. This article provides an overview of how FDA regulates these products. For the purposes of this discussion, this article will use the terms “medical device”, “device” and “product” interchangeably. The article also uses the two terms, “manufacturer” and “developer”, interchangeably.

## Medical device classification and its regulatory implications

The statutory foundation of medical device regulation is a 3-tiered risk-based classification system. Every medical device is classified according to its specific risks to health, from low to high, and each classification level establishes a set of controls from which FDA chooses to best regulate the individual product. See Table 1.

□ Class I: General Controls
□ Class II: Special Controls
□ Class III: Premarket Approval

Table 2 shows the classification of menstrual pads and tampons, menstrual cups and vaginal pessaries. Developers of medical devices are strongly encouraged to explore the CDRH/FDA website (3) and learn more about device classification and the regulatory controls that derive from each class. This website is a good place to start.

**Table 1:** below summarizes the regulatory controls provided by each classification level.

Medical device classification (1)	
Class I: general controls with and without exemptions	*General Controls are a set of regulatory requirements that generally apply to all medical devices. For some medical devices, these are sufficient to ensure their safety and effectiveness. Discussed further below, these general controls include establishment registration and listing, labeling, medical device reporting, 510(k) premarket notification, the investigational device exemption (IDE) regulations that apply to clinical trials, good manufacturing practices (GMPs) as set forth in the Quality Systems regulation, general labeling requirements and medical device reporting (MDR) in the postmarket setting.* *FDA has exempted most Class I devices from the requirement for 510(k) premarket notification.*
Class II: special controls also includes general controls	*When FDA determines that Class I general controls are insufficient to ensure the safety and effectiveness of a type of medical device, the agency assigns that device to Class II. Special Controls are typically device-specific and may include performance standards, postmarket surveillance and/or patient registries, special premarket data requirements, specific labeling requirements, and adherence to designated FDA guidelines.*
Class III: premarket approval	*If FDA determines that Class I and Class II regulatory controls are insufficient to ensure the safety and effectiveness, the agency assigns that device to the highest risk category: Class III Premarket Approval (PMA). The PMA application is a rigorous premarket FDA review process.* *The intravaginal products subject of this issue are not Class III devices, so this article will not spend time on Class III requirements.*

**Table 2:** Classification of intravaginal devices.

The classification of each medical device can be found in the Code of Federal Regulations (CFR), Title 2 (2) 1. Listed below are the classification designations of several intravaginal devices.	
Menstrual pad	Class I: (21 CFR 884.5425 and 884.5435)
Menstrual tampon	Class II: (21 CFR 884.5460 and 884.5470)
Menstrual cup	Class II: (21 CFR 884.5400)
Vaginal pessary	Class II: (21 CFR 884.3575)

With a few narrowly defined exceptions, Class I menstrual pads are exempt from the requirement of 510(k) premarket notification. Class II menstrual cups are also exempt from the 510(k) requirement.

This article will explore how this basic regulatory structure applies to menstrual tampons and vaginal pessaries.

## The 510(k) premarket notification and bringing a menstrual tampon to market

To bring its new product to market, the manufacturer (or developer) of a Class II medical device must submit a *510(k) premarket notification* to the FDA. The essence of such a notification is the identification of a legally-marketed device for comparison, known as a *predicate device*, and a showing that the new device is *substantially equivalent* to the predicate device in terms of safety and effectiveness, with an emphasis on device design and intended use. In making the 510(k) submission, the manufacturer follows the prescribed regulations and FDA guidance, more on that later. If the manufacturer's arguments for substantial equivalence prevail and FDA determines that the new product (in this case a menstrual tampon) is substantially equivalent to the predicate device, then the manufacturer (or product developer) may bring the product to market. However, if FDA finds that the new device design raises new types of questions on safety or effectiveness, or if FDA finds that the indication for use represents a new intended use, then FDA will issue a finding of NOT (emphasis added) substantially equivalent. By statute, a determination of *not substantially equivalent* places the new device into Class III, and a Premarket Approval Application is required. For a tampon, this latter possibility is highly unlikely.

To help the developer of a new menstrual tampon plan for and compile a 510(k) premarket notification, FDA provides a device-specific guidance (dated 2005) that delineates the specific kinds of information and data needed to support the tampon application (4). FDA also provides substantial general guidance for developing its arguments for *substantial equivalence* in the preparation of a 510(k) premarket notification (5). The manufacturer should carefully consider and apply the guidance from both documents, as well as follow the 510(k) regulation (6) itself that spells out the basic format and requirements for a 510(k) submission.

Looking first at FDA's 2005 guidance document for tampon 510(k) submissions, a manufacturer sees that FDA expects the following information:

□ *Description of the Menstrual Tampon*, including the tampon design and dimensions, tampon absorbency, and a full accounting of all component materials of the tampon, including additives.
□ *Potential Risks to Health* Attributed to Use of Menstrual Tampons

The manufacturer is expected to delineate any and all potential risks from tampon use and list the mitigations to address each risk. In Table 3 of its tampon guidance (p. 27) (4), FDA identifies known risks associated with tampon use and provides recommended measures to mitigate those risks. These risks include adverse tissue reaction, vaginal injury, vaginal infection and toxic shock syndrome (TSS). Manufacturers should use this guidance to prepare its own Risk Analysis table, including any other information it may be aware of.

□ *Performance Characteristics* of the Menstrual Tampon

Per its 2005 guidance, FDA expects to see the results from the following performance testing:

○ *Tampon Absorbency* The test methodology for tampon absorbency (also called “syngyna testing”) is specified in 21 CFR 801.430(f). Labeling for tampon absorbency must comply with this regulation. See further below.
○ *Chemical Residues* (testing to show the tampon is free of dioxins and any pesticide and herbicide residues)
○ *Physical testing of String Strength, Fiber Shedding and Tampon Integrity*

□ *Material Safety* Results from Preclinical Toxicological Testing

To determine the types of toxicological testing needed to establish the material safety of a medical device, FDA relies on an international standard, ISO 10993, entitled “Biological Evaluation of Medical Devices – Evaluation and Testing within a Risk Management Process” (7). Use of this standard begins with Part 1 that provides an algorithm for choosing the appropriate toxicological tests for any given medical device, depending on the nature of tissue exposure and the duration of exposure. In 2020, FDA published guidance on how this international standard may be used to support material safety testing (8). In its tampon-specific guidance document, FDA lists the specific tests that are expected (below). The reference in ISO 10993 for the methodology for each toxicological test is given in parenthesis.

○ cytotoxicity (ISO 10993-5)
○ sensitization (ISO 10993-10)
○ vaginal irritation (ISO 10993-10)
○ local effects after implantation (ISO 10993-6)

An experienced, credentialed laboratory is important for the conduct of such testing. Other possible material safety risks such as acute, subchronic and chronic toxicity, reproductive/developmental toxicity and carcinogenicity may be addressed using a risk analysis approach that obviates the need for further testing. A trained toxicologist is necessary for such a risk analysis, as well as for the review and analysis of the results of the toxicological testing that was done.

□ Risk of *Toxic Shock Syndrome (TSS)* and Preclinical *Microbiological Testing*

FDA also expects the manufacturer to evaluate the potential of a menstrual tampon to determine whether it enhances the growth of *Staphylococcus aureus*, increases production of TSS Toxin-1, or alters the growth of normal vaginal microflora. Such testing should be conducted by an experienced laboratory.

□ *Clinical Studies*, *if needed*

Premarket clinical studies are rarely, if ever, needed to support a 510(k) premarket notification for a menstrual tampon. However, if necessary, FDA has provided substantial guidance on the design of clinical studies for medical devices, most notably its 2013 guidance document, entitled “*Design Considerations for Pivotal Clinical Investigations for Medical Devices*” (9). Before initiating a clinical study, manufacturers are strongly encouraged to consult with FDA on the key elements of any such study. FDA also provides a guidance document on how to conduct such early interactions with the agency (10).

□ *Labeling* For menstrual tampons, two labeling regulations apply: (1) a general labeling regulation that applies to all medical devices (11), and (2) a tampon-specific regulation (12) providing exactly what should be contained in tampon labeling about tampon absorbency and the risk of toxic shock syndrome (TSS). This latter regulation also provides the methodology for the absorbency test (sometimes called “syngyna test”) to determine tampon absorbency for the labeling.

In preparation for its submission of a 510(k) premarket notification, a manufacturer should then compile and organize all of the above information—both descriptive information, as well as results and analysis from the various studies—to cogently demonstrate that the new tampon is *substantially equivalent* to a legally marketed menstrual tampon already on the US market.

To further help medical device developers, FDA posts information on each 510(k) decision it makes, including the SE determination letter, an indications for use sheet, and—typically—a brief summary of information supporting the 510(k) submission. For regulatory perspective, it can be very useful to review that historical information (13).

In its 2014 510(k) guidance on how to evaluate *substantial equivalence*, FDA explains how such arguments are made. To help manufacturers with this, the guidance document includes a substantial equivalence logic flowchart to show how the decision-making works (diagram below) (5).

**Figure 1:**
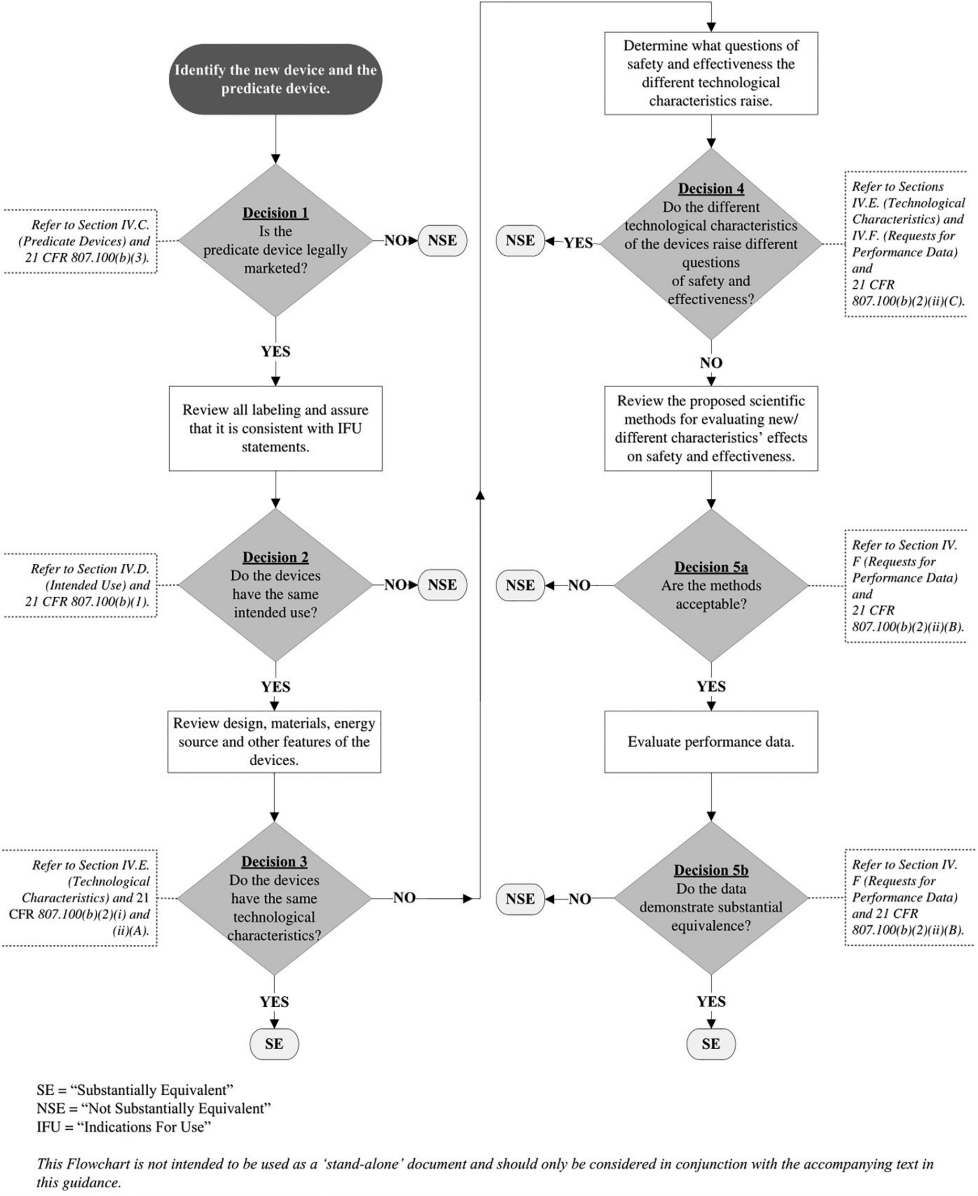


Regarding use of the flowchart in Figure 1, the FDA guidance emphasizes that the “…flowchart is NOT [*emphasis added*] intended to be used as a ‘stand-alone’ document and should only be considered in conjunction with the accompanying text in this guidance.” The flowchart and accompanying text should include all appropriate references to the context of each critical decision point.

When the 510(k) premarket notification is received, the statute provides 90 days for the agency to complete its review. FDA may break this 90-day review time into parts, putting the document “on hold” to ask certain follow-up questions. The manufacturer should carefully consider the questions and provide full response. FDA contact information is provided, and if anything is unclear, manufacturers are encouraged to connect directly with the reviewer.

## Bringing a vaginal pessary to market

Bringing a vaginal pessary to market would follow a regulatory pathway similar to that described above for menstrual tampons. Again, the manufacturer (or developer) must submit a *510(k) premarket notification* to the FDA, following the prescribed regulations and guidance. The submission must provide information showing that the new vaginal pessary is *substantially equivalent*, in terms of safety and effectiveness, to another similar pessary already legally on the market, the so-called *predicate device*.

While FDA has not produced a device-specific guidance document on vaginal pessaries, many of the same principles used for the tampon apply to the vaginal pessary. The general guidance for developing arguments for substantial equivalence, discussed above, apply here (14). As with the tampons, the manufacturer should carefully consider this guidance and follow the 510(k) regulation (15) itself that spells out the basic format and requirements for a 510(k) submission.

When preparing a 510(k) premarket notification for a new vaginal pessary and considering the types of information needed to support an argument for substantial equivalence, the manufacturer (or developer) must also consider the intended use of the pessary. Unlike the tampon, a vaginal pessary—depending on its design and the manufacturer's intention—may be used to manage urinary incontinence and/or pelvic organ prolapse. Again, depending on design and manufacturer intention, a new pessary might be available over-the-counter or as a prescription-only device. It might require physician evaluation, need for fitting, insertion and removal, or it might be designed so that the user could insert it and remove it herself. The pessary might be intended as single-use disposable device, for a short period of time, or for longer-term use, reusable with instructions for cleaning. When building the argument for substantial equivalence, the manufacturer must consider all of these device features in the context of the 510(k) SE flowchart (Figure 1), especially as it relates to the predicate device, the indication for use and intended use and whether that might trigger the need for clinical data.

Using the tampon 510(k) submission (described above) as a model, a manufacturer should follow a similar path and can expect that FDA will expect the following information for a new pessary:

□ *Description of the Vaginal Pessary*, including the pessary design and dimensions, insertion features, and a full accounting of all component materials, including additives.
□ *Potential Risks to Health* attributed to Use of Vaginal Pessary

FDA has not developed a device-specific guidance document on vaginal pessaries, so the manufacturer must conduct its own independent risk analysis, delineating all potential risks from pessary use and the mitigations to address each risk. Many of these potential risks are obvious, e.g., adverse tissue reaction, vaginal injury, vaginal infection and toxic shock syndrome (TSS). In its independent risk analysis, manufacturers should include any other information it may be aware of.

□ *Performance Characteristics* of the Vaginal Pessary

Considering FDA's general guidance, as well as the types of information FDA expects for tampons, manufacturers should test the performance specifications of the pessary in the context of the risk analysis and identified mitigations. Such physical bench testing might include:

◾ applicator (if any) insertion and removal force
◾ core properties of the finished pessary and applicator (if any), e.g., dimensions, weight, material properties, compression characteristics
◾ removal string (if any) detachment force and string integrity

□ *Material Safety* | Results from Preclinical Toxicological Testing

As with the tampon, the manufacturer of a new vaginal pessary should apply the international standard, ISO 10993, Part 1 (16), to determine the appropriate toxicological tests that should be undertaken. For the vaginal pessary, considering its mucosal contact, possible indirect blood contact for long-term and/or repeated use duration, the following tests are recommended, at a minimum:

○ cytotoxicity (ISO 10993-5)
○ sensitization (ISO 10993-10)
○ vaginal irritation (ISO 10993-10)
○ local effects after implantation (ISO 10993-6)

At this stage of the process, a trained toxicologist should be engaged to evaluate the pessary and determine the full battery of toxicological testing. An experienced, credentialed laboratory is important for the conduct of such testing. As with the tampon, other possible material safety risks such as acute, subchronic and chronic toxicity, reproductive/developmental toxicity and carcinogenicity may be addressed using a risk analysis approach that obviates the need for further testing. A trained toxicologist is necessary for such a risk analysis, as well as for the review and analysis of the results of the toxicological testing that was done.

□ Risk of *Toxic Shock Syndrome (TSS)* and Preclinical *Microbiological Testing*

FDA also expects the manufacturer of a new vaginal pessary to determine its potential for enhancing the growth of *Staphylococcus aureus*, or increasing production of TSS Toxin-1, or altering the growth of normal vaginal microflora. Such testing should be conducted by an experienced laboratory; appropriate expertise should be used in selecting such a laboratory.

□ *Clinical Studies*, *if needed*

Typically, no clinical studies will be needed to support a 510(k) for a vaginal pessary. However, if new use features are introduced, e.g., OTC/self-administration, it may be necessary to conduct small clinical validation studies to evaluate label comprehension, self-selection, and self-administration. And, depending on the intended use of the pessary, the primary study endpoint of such a clinical study might be prolapse stage (for pelvic organ prolapse) or number of incontinence pads needed (for urinary incontinence). The manufacturer should consider whether an early consultation with FDA staff would be helpful to ensure the clinical study design will answer the questions that the agency is likely to have. See FDA guidance on Q-submissions (10). It might also be helpful to re-visit FDA's 2013 general guidance document on the design of clinical studies for medical devices, discussed earlier.

□ *Labeling* While there is no device-specific labeling regulation for vaginal pessaries, manufacturers must comply with the general labeling regulation for medical devices (21 CFR 801) (11).

If and when a manufacturer receives a decision letter from FDA with a determination of substantial equivalence, by statute that is—in effect—regulatory permission to market the device in the US.

## 510(k) premarket notification | administrative items to consider

◾ Format of a 510(k) submission. FDA has provided general guidance on the basic format of a 510(k) submission (17).
◾ 90-day statutory review timeline. By statute, FDA has 90-days to complete its review of a 510(k) submission. Depending on questions the FDA review team may have, the 90-days may be split between the initial review and a subsequent review of FDA questions.
◾ Submission of a 510(k) and correspondence with FDA. For FDA to successfully complete its review, the manufacturer must carefully and thoroughly respond to all FDA concerns in a timely manner. If there is any uncertainty about what is needed, the manufacturer is encouraged to contact the FDA review team and clarify what FDA is looking for.
◾ User fees. Submission of a 510(k) is governed by the Medical Device User Fee Amendments (MDUFA) (18). In FY2023, the user fee for a 510(k) submission is $19,870USD ($4,967USD for certified small business).

It also important to recognize that FDA employs a comprehensive set of postmarket controls to ensure the continued safety and effectiveness of the device in the market and use settings. Perhaps the most important are good manufacturing practices (GMPs), as spelled out in the Quality Systems regulation (21 CFR Part 820). Other relevant controls include annual registration of device manufacturing facilities, listing of devices, labeling, mandatory device reporting (adverse events) and prohibitions against misbranding (false or misleading in any particular) and adulteration (e.g., corrupted or debased by incorporating an impure or spurious substance). While these regulatory controls are critical to the continued safety and effectiveness of menstrual tampons and vaginal pessaries, this article will not go into the details of those controls.

## Conclusion

In summary, in the United States, menstrual tampons and vaginal pessaries are medical devices, subject to a set of premarket and postmarket regulations. Practicing clinicians are often the idea-source for innovative improvements of these products. When working with a product development team, these clinicians should be cognizant of the regulatory environment and be prepared to help their teams accordingly. It is hoped that this article will serve as a useful starting point for clinicians embarking on a program contributing to a 510(k) premarket notification for a menstrual tampon or a vaginal pessary. Clinicians new to such programs seeking marketing authorization from FDA would be well advised to work closely with the regulatory specialist on the product development team before beginning on any clinical trials or initiating contact with FDA personnel.

## Data Availability

The original contributions presented in the study are included in the article/Supplementary Material, further inquiries can be directed to the corresponding author.
